# Bilateral simultaneous knee arthroplasty shows comparable early outcome and complication rate as staged bilateral knee arthroplasty for patients scored ASA 1–3 if performed by a high-volume surgeon: a retrospective cohort study of 127 cases

**DOI:** 10.1007/s00402-023-05078-4

**Published:** 2023-10-09

**Authors:** Stephanie Kirschbaum, Robert Hube, Carsten Perka, Michael Najfeld

**Affiliations:** 1 Centre for Musculoskeletal Surgery, Charité-University Hospital Berlin, Charitéplatz 1, 10117 Berlin, Germany; 2 OCM Orthopädische Chirurgie München, Steinerstraße 6, 81369 Munich, Germany

**Keywords:** Bilateral simultaneous knee arthroplasty, One-stage knee arthroplasty, Two-stage knee arthroplasty, Staged knee arthroplasty, Complications

## Abstract

**Background and purpose:**

The study compares early outcomes after simultaneous and staged knee arthroplasty in patients with bilateral knee osteoarthritis (OA) to evaluate whether simultaneous bilateral TKA shows comparable early outcomes and complication rates to staged bilateral TKA.

**Methods:**

A retrospective cohort study including all patients scheduled for primary TKA for bilateral knee OA (*n* = 127) was conducted. Patients received either simultaneous (*n* = 53, 41.7%) or staged (*n* = 74, 58.3%) bilateral TKA by a single, high-volume surgeon—depending on their individual preference. Demographic data, haemoglobin drop (Hb), length of stay (LOS), operation time, 30-day complication rate and achievement of rehabilitation key points were evaluated.

**Results:**

There was no difference between the groups concerning age, sex, BMI or complication rate. ASA scoring was better in the simultaneous group [2.2, (15.1% ASA 1, 49.1% ASA 2, 35.8% ASA 3) vs. 2.4 (2.7% ASA 1, 51.4% ASA 2, 45.9% ASA 3)]. Average LOS was 7.8 ± 2.1 days for simultaneous TKA, 7.4 ± 1.7 days for single procedure of staged group (*p* < 0.453) and 14.7 ± 3.1 days if combined (*p* < 0.001). Cumulative Hb loss was significantly higher in the staged group (3.8 ± 1.2 g/dl vs. 2.4 ± 0.8 g/dl, *p* < 0.001). Detailed comparison of early outcome parameters between staged and simultaneous procedure depending on ASA score only revealed slightly slower assessment of stairs (*p* < 0.001) and increased Hb drop per surgery in case of simultaneous procedure (*p* < 0.011) if ASA score was ≥ 2. Only patients scored ASA 3 demonstrated a significant longer LOS per procedure in simultaneous group (8.5 ± 2.4 vs.7.3 ± 1.6 days, *p* = 0.034).

**Interpretation:**

Simultaneous bilateral TKA results in comparable early outcome and complication rate than staged bilateral procedure—even for patients scored ASA 3.

**Level of evidence:**

Level IV.

## Introduction

87% of patients scheduled for TKA also demonstrate radiographic evidence of osteoarthritis on the contralateral knee [9]. Patients with unilateral knee osteoarthritis already demonstrate abnormal loading in the unaffected knee, which may accelerate disease in other joints due to changes in the gait [21]. If there is no “unaffected” knee, patients regularly experience quick decrease in their physical function and experience an increase in overall pain [6, 16, 23]. To date, there is no consensus regarding the optimal surgical strategy for patients presenting with severe bilateral knee pain and OA. Whilst staged bilateral TKA represents the gold standard and seems to have a lower complication risk [2, 13, 18, 20], patients regularly ask for simultaneous bilateral TKA, as it provides some organisational comfort: one anaesthesia, one hospital stay, one rehabilitation period [4, 31], one period of impaired mobility [3] and therefore only one period of loss of independence. Furthermore, simultaneous bilateral TKA results in lower costs for the health care system [12, 24, 30].

As there are several reports about higher mortality, risk of transfusion and thromboembolic events [13, 18, 20], many authors do not recommend performing simultaneous bilateral TKA [2, 22]. Unfortunately, most of the studies lack information about the American Society of Anesthesiologists (ASA) scores or lack any evaluation of early or mid-term functional outcomes [8, 13, 18, 20]. Furthermore, their comparability is often limited due to different surgical techniques and rehabilitation protocols.

Therefore, the general conclusions concerning risks and safety seem to be vague. Therefore, the aim of this recent study was to compare the complication rate, blood loss and early functional outcome associated with simultaneous bilateral TKA compared to staged bilateral TKA performed by a single, high-volume arthroplasty surgeon. Furthermore, the impact of ASA score on outcome parameters and complication rate was evaluated.

## Patients and methods

**Fig. 1:**
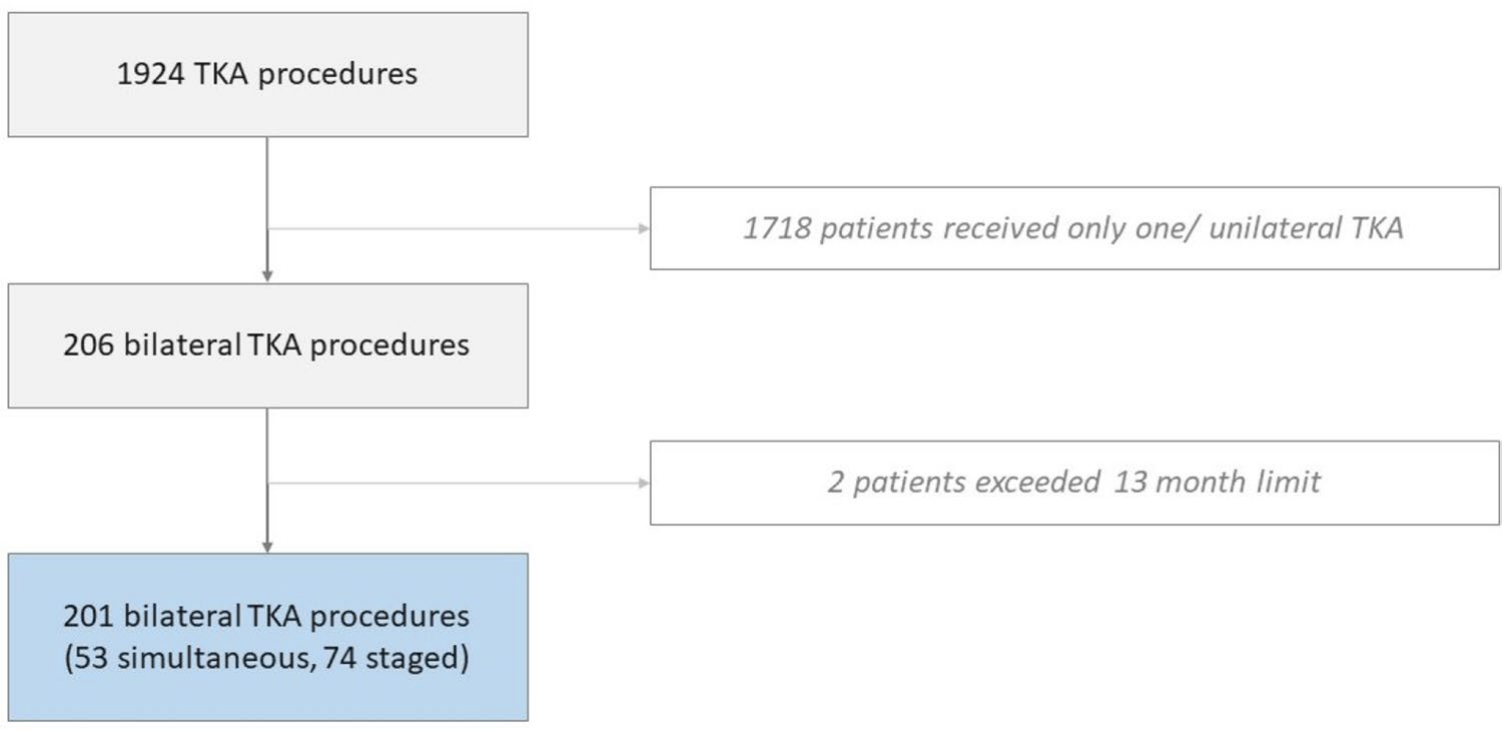
Enrolment of patients in the current study

The study protocol was submitted to the local ethics committee (number: 21004). The institutional database was retrospectively analysed for patients undergoing primary TKA by a single high-volume surgeon between January 2015 and December 2020 (*n* = 1924). In this retrospective, single-centre cohort study, only patients with simultaneous or staged bilateral TKA procedures were included. All patients were offered simultaneous as well as staged procedures. As this shared decision-making process determined whether the patient would undergo simultaneous or staged procedures, there was no further randomisation or matching of the groups. In the staged group, the most symptomatic knee was always treated first. Staged bilateral TKA was defined as 2 separate surgeries within 13 months. Patients who exceeded this time range were excluded from the study (*n* = 2). In total, *n* = 127 patients (*n* = 201 procedures) were included in the study. The patient cohort was divided into 2 groups: simultaneous (*n* = 53, 41.7%) versus staged bilateral TKA group [*n* = 74, 58.3% (Fig. 1)]. No exclusion was made due to age, BMI, ASA score or the complexity of osteoarthritis (e.g. severe varus or valgus alignment, posttraumatic). Every patient received Persona Knee (Fa. Zimmer).

### TKA protocol

All surgeries were performed by the same high-volume surgeon using a medial parapatellar approach. Simultaneous TKA procedures were performed sequentially—one knee after another within one surgical session. Implant design was chosen based on anatomy, stability and bone quality. All departments (anaesthesia, surgery, nursing and physiotherapy) had implemented protocols, including the use of tranexamic acid (1 g intravenous preoperatively and 3 g intraarticular after capsular suture) and opioid-sparing analgesia with additional acetaminophen and non-steroid anti-inflammatory drugs (NSAIDs), depending on the patient’s pain level and preexisting comorbidities.

The postoperative rehabilitation regimens were independent from simultaneous or staged procedures and started on the day of surgery: first, mobilisation of the patient was performed under the supervision of a physiotherapist and included transfer and gait training using a walker or crutches, as well as active-assisted range of motion exercises. Full weight-bearing was allowed. The two key points of inpatient rehabilitation included unaccompanied walking within the ward and, as soon as possible, being able to access stairs.

### Demographic characteristics and outcome parameters

The patient records were assessed for patient-related and demographic measures, including age (years), sex, body mass index (BMI kg/m^2^) and ASA score. Furthermore, postoperative haemoglobin drop (Hb), need for red blood cell transfusion, length of stay (LOS) and operation time were assessed. The haemoglobin drop was analysed by comparison of the preoperative blood haemoglobin level (g/dl) to the blood haemoglobin level the day after surgery. The length of stay started with inpatient treatment the day before surgery when surgical and anaesthesiologic evaluations of the patient were performed and ended with patient discharge. The discharge criteria were a dry wound, ability to walk unaccompanied in the ward and the assessments for safely accessing stairs. Time needed to reach the mentioned rehabilitation key points [unaccompanied walking (milestone floor) and assessment of stair climbing (milestone stair)] were also evaluated. To achieve those milestones, the patients were supervised by our inpatient physiotherapists. Operation time was defined by the incision of the skin and finish of the wound closure and added for left and right knee replacement for statistical comparison.

Medical (e.g. symptomatic deep vein thrombosis or thromboembolism) as well as surgical (e.g. hematoma, wound-healing disorders, infection, fracture) complications within 6 weeks after surgery were documented as well. Every patient received follow-up of 6 weeks after surgery at the institutional outpatient department for identification of early complications and radiographic control.

### Statistical analysis

Statistical analysis was performed using SPSS Version 26 (SPSS Inc., Chicago, IL, USA). Data were expressed as the mean ± SD. The Shapiro‒Wilk test was used to test the Gaussian distribution. Next, comparison of the parametric data was performed with the Mann‒Whitney test or *t* test. The Chi-square test was used to compare the distribution of ordinal variables. The Kruskal‒Wallis test and the ANOVA were used to identify differences in metric parameters between patients with ASA scores 1–3.

Correlations between the metric and ordinal parameters were calculated via the Kendall tau-*b* test, and correlations between the metric parameters were calculated with a Pearson correlation test. The significance level of all tests was 5% (2-sided). In case *t* test was used, 95% confidence interval was additionally reported.

## Results

A total of 127 patients (63 women, 64 men) were included in the study. Fifty-three patients received simultaneous TKA (41.7%) and 74 patients received staged TKA (58.3%), for a total of 201 TKA procedures. The average time between TKA and the staged procedure was 7.9 ± 3.2 months [2.6–13.3]. The overall age was 68.6 ± 8.6 [48–87] years. The overall BMI was 28.2 kg/m^2^ ± 4.7 [18.9–43.2]. The average ASA score was 2.4 ± 0.6 [1–3]. Only ASA scoring showed significant differences between the simultaneous and staged groups (Table 1).

**Table 1:** Comparison of demographic data between the simultaneous and staged bilateral TKA groups

	Simultaneous bilateral TKA (*n* = 53)	Staged bilateral TKA (*n* = 74)	Significance
Age (y)	69.6 ± 8.0 [49–84]	68.2 ± 8.8 [48–87]	0.308 [CI − 1.31–4.12]
Sex (women/men, %)	41.5/58.5	55.4/44.6	0.122
BMI (kg/m^2^)	28.1 ± 4.6 [18.9–43.2]	28.2 ± 4.7 [20–42.7]	0.833
Average ASA score and distribution	2.2 ± 0.7 [1–3] ASA 1: 15.1% ASA 2: 49.1% ASA 3: 35.8%	2.4 ± 0.6 [1–3] ASA 1: 2.7% ASA 2: 51.4% ASA 3: 45.9%	0.033

*ASA* American Society of Anesthesiologists, *BMI* Body Mass Index, *TKA* Total Knee Arthroplasty

### Surgical parameters

The operation time in the simultaneous bilateral TKA group was 85.3 ± 16.8 min [61–120]. The average operation time of one TKA surgery in the staged bilateral TKA group was 40.1 ± 8.4 min [25–72], and the cumulative operation time in the staged group was 80.2 ± 13.5 min [57–138] per patient. No significant differences in the cumulative operation time were found between the simultaneous and staged procedures (*p* = 0.149).

Whilst the average Hb loss in the simultaneous group was 2.4 ± 0.8 g/dl [1–4], the average Hb loss in the staged group was 1.9 ± 0.7 g/dl [0.2–3.7] per surgery (*p* = 0 < 0.001, 95% CI 0.288 to 0.734). The cumulative Hb loss was significantly higher in the staged group (3.8 ± 1.2 g/dl [0.8–6.9], *p* < 0.001, 95% CI − 1.78 to − 1.02). The transfusion rate for both groups was 0%.

### Length of stay and rehabilitation

Whilst the average LOS was 7.8 ± 2.1 days [4–16] for simultaneous bilateral TKA, the combined LOS was 14.7 ± 3.1 days [9–30] for staged bilateral TKA, which was significantly longer (*p* < 0.001). The LOS for single TKA in the staged procedure was 7.4 ± 1.7 days [4–17] (*p* < 0.453).

No significant difference was found between simultaneous and staged bilateral TKA concerning free walking in the ward, whereas the simultaneous TKA groups needed longer to be able to access stairs (Table 2).

**Table 2:** Comparison of key points concerning inpatient mobilisation

	Simultaneous bilateral TKA	Staged bilateral TKA	Significance
Free walking at ward [at day]	2.9 ± 1.5 [1–9]	2.4 ± 0.8 [1–5]	0.055
Exercising stair climbing [at day]	4.4 ± 1.3 [3–9]	3.5 ± 1 [2–7]	< 0.001

*TKA *total knee arthroplasty

### Complications

Whereas 1.5% (*n* = 3, 2 × arthrofibrosis requiring arthroscopic arthrolysis, 1 × superficial wound-healing disorder) of these complications occurred after simultaneous bilateral TKA, 0.5% (*n* = 1, periprosthetic infection requiring debridement, irrigation and insert exchange) occurred after staged bilateral TKA (*p* = 0.085).

No deaths and no symptomatic thromboembolic events occurred within 30 days of surgery for all patients.

### Influence of ASA score on surgical and functional outcomes

Independent of simultaneous or staged procedures, there were no significant differences in LOS, operation time, average Hb drop per surgery or achievement of rehabilitation key markers between the patients with ASA scores of 1, 2 or 3 (Table 3). Only age and BMI correlated with ASA score (age: Kendall tau-*b* 0.158, *p* = 0.006, BMI: Kendall tau-*b* 0.543, *p* < 0.001).

**Table 3:** Demographic and outcome parameters depending on ASA score

	ASA Score 1 (*n* = 12)	ASA Score 2 (*n* = 102)	ASA Score 3 (*n* = 87)	Significance
Age [years]	74.3 ± 4.8 [65–80]	69.2 ± 8.7 [48–87]	67 ± 8.5 [49–85]	*p* = 0.013
BMI [kg/m^2^]	24.0 ± 6.1 [18.9–42.7]	26.1 ± 3.0 [20.4–42.7]	31.3 ± 4.2 [20.0–43.2]	*p* < 0.001
LOS [days]	7.2 ± 0.7 [6–9]	7.4 ± 1.9 [4–17]	7.6 ± 1.8 [4–15]	*p* = 0.922
Operation time per surgery [min]	71.1 ± 31.5 [30–112]	49.8 ± 20.8 [25–120]	52 ± 22.9 [25–120]	*p* = 0.177
Hb drop per surgery [mg/dl]	2.4 ± 0.8 [1.1–3.8]	2.0 ± 0.7 [0.2–4.0]	2.1 ± 0.7 [0.6–4.0]	*p* = 0.114
Free walking at ward [at day]	2.3 ± 1.0 [1–4]	2.5 ± 1.1 [1–9]	2.6 ± 1.1 [1–9]	*p* = 0.409
Exercising stair climbing [at day]	3.5 ± 0.9 [3–6]	3.7 ± 1.2 [2–9]	3.8 ± 1.1 [2–7]	*p* = 0.668
Complication rate [%]	8.3	1	2.3	0.217

*ASA* American Society of Anesthesiologists, *BMI* Body Mass Index, *Hb* Haemoglobin, *LOS* Length of Stay

Concerning detailed comparison of outcome parameters between staged and simultaneous procedure, within each ASA scoring (Table 4), patients scored ASA ≥ 2 demonstrated slightly slower assessment of stairs and increased Hb drop in case of simultaneous procedure. There were no differences concerning free walking at ward and complication rate. Only patients scored ASA 3 demonstrated a significant longer LOS in simultaneous group. This trend was not found in patients scored ASA 1 or 2.

**Table 4:** Comparison of key points concerning perioperative parameters and inpatient mobilisation between simultaneous and staged group depending on ASA scoring

	Simultaneous bilateral TKA	Staged bilateral TKA	Significance
**ASA Score 1 (*n* = 12)**			
Average operation time per surgery [min]	90.4 ± 16.7 [68–112]	32.5 ± 2.6 [30–36]	< 0.001 [95% CI 38.7–77.0]
Hb drop per surgery [g/dl]	2.5 ± 1.0 [1.1–3.8]	2.3 ± 0.4 [1.9–2.7]	0.670 [95% CI − 0.92–1.37]
Cumulative Hb drop [g/dl]	2.5 ± 1.0 [1.1–3.8]	5.1 ± 0.2 [4.9–5.2]	0.044
LOS per surgery [days]	6.9 ± 0.4 [6, 7]	7.8 ± 1.0 [8, 9]	0.154
Cumulative LOS [days]	6.9 ± 0.4 [6, 7]	18 ± 4.2 [15–21]	0.044
Free walking at ward [at day]	2.4 ± 0.9 [1–4]	2 ± 1.2 [1–3]	0.683
Exercising stair climbing [at day]	3.8 ± 1.0 [3–6]	3 ± 0.1.0 [3–3]	0.214
Complications [%] of all ASA 1	8.3 [n = 1]	0	0.460
**ASA Score 2 (*n* = 102)**			
Average operation time per surgery [min]	80.6 ± 15.9 [61–120]	39.2 ± 7.4 [25–72]	< 0.001
Hb drop per surgery [g/dl]	2.3 ± 0.8 [1.1–4]	1.9 ± 0.7 [0.2–3.7]	0.011 [95% CI − 0.09–0.74]
Cumulative Hb drop [g/dl]	2.3 ± 0.8 [1.1–4]	3.7 ± 1.3 [0.8–6.9]	< 0.001
LOS per surgery [days]	7.5 ± 2.1.0 [4–16]	7.4 ± 1.9 [4–17]	0.736
Cumulative LOS [days]	7.4 ± 2.1.0 [4–16]	14.9 ± 3.5 [9–30]	< 0.001
Free walking at ward [at day]	3.0 ± 1.5 [1–9]	2.5 ± 0.9 [1–5]	0.082
Exercising stair climbing [at day]	4.4 ± 1.5 [3–9]	3.4 ± 1.0 [2–7]	< 0.001
Complications [%] of all ASA 2	1 [n = 1]	0	0.086
**ASA Score 3 (*n* = 87)**			
Average operation time per surgery [min]	89.5 ± 17 [61–120]	41.5 ± 9.2 [25–72]	< 0.001
Hb drop per surgery [g/dl]	2.6 ± 0.8 [1–4]	1.9 ± 0.7 [0.6–3.7]	0.001 [95% CI 0.28–0.98]
Cumulative Hb drop [g/dl]	2.6 ± 0.8 [1–4]	3.9 ± 1.1 [1.5–5.7]	< 0.001
LOS per surgery [days]	8.5 ± 2.4 [6–15]	7.3 ± 1.6 [4–12]	0.034
Cumulative LOS [days]	8.5 ± 2.4 [6–15]	14.4 ± 2.4 [10–19]	< 0.001
Free walking at ward [at day]	3.1 ± 1.6 [1–9]	2.5 ± 0.8 [1–5]	0.153
Exercising stair climbing [at day]	4.5 ± 1.3 [3–7]	3.5 ± 1 [2–7]	< 0.001
Complications [%] of all ASA 3	6.3	3.7	0.329

*ASA* American Society of Anesthesiologists, *Hb* Haemoglobin, *LOS* length of stay

## Discussion

Simultaneous bilateral TKA procedures seem to be an attractive option in cases of bilateral osteoarthritis of the knee, as they decrease the rehabilitation time for the patient as well as the overall costs for the health care system [11, 15, 26]. However, despite modern fast-track concepts, the current literature reports a significantly higher incidence of thromboembolic events, transfusion rate and mortality for simultaneous bilateral TKA [7, 8, 20, 26, 29]. The small number of prospective studies, the existing selection bias (including only ASA score 1 or 2), the different surgical and perioperative management protocols and the missing evaluations of especially early functional outcomes limit the conclusions of many existing studies. Therefore, the recent study is the first to demonstrate noninferiority concerning early rehabilitation outcomes as well as the risk of thromboembolic events or the 30-day complication rate of simultaneous bilateral TKA performed by a single high-volume surgeon.

The current meta-analysis reported higher mortality in simultaneous bilateral TKA (OR 2.24) [8, 18, 20], which might be the result of a higher risk of thromboembolic events [1, 20, 29]. In addition to surgical procedures, a higher ASA score (3 and 4) or Charleston Index impacts the complication risk [29, 32]. Therefore, patients staged ASA 3 or 4 often receive staged bilateral TKA, resulting in a certain selection bias.

Finding significantly higher mortality rates and thromboembolic events in the simultaneous bilateral TKA group seems surprising, as patients in the simultaneous group are usually in better general condition [8, 18, 20]. On the other hand, patients with severe comorbidities (ASA scores of 3 and 4) usually receive more intense medical care and are more likely to already take oral anticoagulation medications regularly [5]. Therefore, the lower thromboembolic event rate in the staged bilateral TKA group might be the result of a higher percentage of therapeutic instead of prophylactic doses of anticoagulation, keeping in mind the usually higher ASA scoring in the staged bilateral group. If the ASA score was evaluated and comparable between simultaneous and staged bilateral TKA, no higher incidence of thromboembolic events was reported in the literature [27]. This study also showed a similar ASA distribution without any differences in complication rates and furthermore no increasing complication rate with increasing ASA scoring. However, most of those meta-analyses and studies unfortunately did not report ASA scoring or patients’ comorbidities [8, 13, 18, 20]. This lack of information makes it difficult to interpret the results without bias.

In addition to ASA score, surgical and rehabilitation protocols also influence thromboembolic risk: Fast-track concepts presented a lower risk of thromboembolic events [14]. Therefore, early mobilisation represents a major protective factor, and comparison of early rehabilitation progress seems crucial for evaluation and interpretation of the perioperative complication rate. The fact that the recent study did not find any significant differences concerning general mobilisation when the patient is on the ward (free walking) between simultaneous and staged TKA—independent from ASA scoring—might be the reason that there was no increased incidence of thromboembolic events. Additionally, the short operation time and the great surgical experience represented in our study might explain the low level of overall complications [17, 25]. The fast operation time might also explain why there were no transfusions necessary in the recent patient cohort compared to the transfusion rates of staged (1.1–8.1%) or simultaneous (7.2–40.8%) bilateral TKA in the literature [7, 8, 11].

Even though achieving free walking whilst in the ward showed no significant difference between staged and simultaneous bilateral TKA, the ability to access stairs took one day more in the simultaneous bilateral TKA group. The latter was especially seen in patients scored ASA ≥ 2. As range of motion is usually painfully inhibited immediately after surgery, going up and down stairs, which requires more flexion of the knee joint, seems consequently more impaired than plain walking. Comparison not only of early but also of mid-term outcomes in literature showed no significant difference in the clinical or the patient-reported outcomes between simultaneous and staged bilateral groups [26]. However, the simultaneous group only needed one day longer to achieve this key point of inpatient rehabilitation, which did not lead to a longer LOS of the simultaneous group (per stay). Only patients scored ASA 3 demonstrated 1 day longer LOS, which nevertheless represents an average benefit of 6 days compared to staged procedure. Different studies have confirmed a shorter overall LOS in the simultaneous bilateral TKA group [11, 20, 26, 32] as well as a reduced period of impairment for the patient, resulting in a reduced financial burden for the health care system [11]. On the other hand, Sobh et al. noted that reduced costs of LOS and rehabilitation are equalised by increased costs for medical treatment of higher complication rates [15, 28]. Yoon et al. reported that an increased complication rate was found only in patients rated as ASA 3 or 4 or aged older than 70 [32]. Therefore, patient selection, as well as surgeon experience, and quick mobilisation seem to be key points in saving simultaneous bilateral TKA procedures.

The recent study has several limitations. First, the cohorts were not matched for age, BMI or ASA score due to the retrospective study design. This might lead to a selection bias, as the simultaneous group showed slightly better ASA score. However, no exclusion was made due to age, BMI, ASA score or the complexity of osteoarthritis (e.g. severe varus or valgus alignment, posttraumatic). The range of the operation time implicates that no patient was excluded due to complexity of TKA procedure which certainly represents a strength of the recent study. Furthermore, the decision of simultaneous or staged procedure was based on the patients’ informed decision—not on ASA scoring or age. Additionally, a detailed analysis concerning influence of ASA scoring on overall outcome as well as separated outcome was performed to overcome the lack of matching. Another limitation is that the occurrence of complications was only evaluated within 6 weeks after surgery, which might cause the authors to miss later complications, such as surgical-site or peri-prosthetic joint infections. As surgery-related acute PPI occurs usually within the first 4–6 weeks after surgery and should therefore been assessed within the follow-up of 6 weeks, this possible limitation seems negligible [10]. As most surgery-associated complications, such as DVT or thromboembolism, occur within the early rehabilitation period (first 7 days [19]), most of those complications should have been assessed too, as average LOS and therefore clinical control in this study was 7 days.

## Conclusions

Simultaneous bilateral TKA represents a successful procedure in patients with bilateral symptomatic knee OA that is not associated with a higher incidence of red blood cell transfusion or complications if performed by an experienced high-volume surgeon in an adequate setting with early mobilisation. Instead, it offers reduced LOS with equal early rehabilitation, which favours patients regaining autonomy and reduces the financial burden of the health care system. Patients scored ASA ≥ 2 also seem to profit from a simultaneous procedure in a specialised setting concerning cumulative LOS and Hb drop but demonstrate slightly demonstrated higher blood loss per procedure as well slight delay in assessment of stairs (1 day). No differences in free walking at ward or complication rate were seen.

## Data Availability

The data that support the findings of this study are available from the corresponding author (S.K) upon reasonable request.

## Abbreviations

ASA: American Society of Anesthesiology
BMI: Body mass index
DVT: Deep venous thrombosis
Hb: Haemoglobin
LOS: Length of stay
NSAIDs: Nonsteroidal anti-inflammatory drugs
OR time: Operation time
OCM: Orthopädische Chirurgie München
POD: Postoperative day
TKA: Total knee arthroplasty

## References

[1] Abdelaal MS, Calem D, Sherman MB, Sharkey PF (2021). Short interval staged bilateral total knee arthroplasty: safety compared to simultaneous and later staged bilateral total knee arthroplasty. J Arthroplasty.

[2] Almaguer AM, Cichos KH, McGwin G, Pearson JM, Wilson B, Ghanem ES (2019). Combined total hip and knee arthroplasty during the same hospital admission: is it safe?. Bone Joint J.

[3] Baczkowicz D, Skiba G, Czerner M, Majorczyk E (2018). Gait and functional status analysis before and after total knee arthroplasty. Knee.

[4] Berg U, Berg M, Rolfson O, Erichsen-Andersson A (2019). Fast-track program of elective joint replacement in hip and knee-patients' experiences of the clinical pathway and care process. J Orthop Surg Res.

[5] Blasius FM, Laubach M, Andruszkow H, Lubke C, Lichte P, Lefering R (2021). Impact of anticoagulation and antiplatelet drugs on surgery rates and mortality in trauma patients. Sci Rep.

[6] Dawson J, Linsell L, Zondervan K, Rose P, Randall T, Carr A (2004). Epidemiology of hip and knee pain and its impact on overall health status in older adults. Rheumatology (Oxford).

[7] Erossy M, Emara AK, Zhou G, Kourkian S, Klika AK, Molloy RM (2022). Simultaneous bilateral total knee arthroplasty has higher in-hospital complications than both staged surgeries: a nationwide propensity score matched analysis of 38,764 cases. Eur J Orthop Surg Traumatol.

[8] Fu D, Li G, Chen K, Zeng H, Zhang X, Cai Z (2013). Comparison of clinical outcome between simultaneous-bilateral and staged-bilateral total knee arthroplasty: a systematic review of retrospective studies. J Arthroplasty.

[9] Gunther KP, Sturmer T, Sauerland S, Zeissig I, Sun Y, Kessler S (1998). Prevalence of generalised osteoarthritis in patients with advanced hip and knee osteoarthritis: the Ulm Osteoarthritis Study. Ann Rheum Dis.

[10] Hansen E, Tetreault M, Zmistowski B, Della Valle CJ, Parvizi J, Haddad FS (2013). Outcome of one-stage cementless exchange for acute postoperative periprosthetic hip infection. Clin Orthop Relat Res.

[11] Hou JF, Hu C, Zhang Y, Tian LQ, Liu YZ, Zhang C (2021). Cost analysis of staged versus simultaneous bilateral total knee and hip arthroplasty using a propensity score matching. BMJ Open.

[12] Houdek MT, Wyles CC, Watts CD, Wagner ER, Sierra RJ, Trousdale RT (2017). Single-anesthetic versus staged bilateral total hip arthroplasty: a matched cohort study. J Bone Joint Surg Am.

[13] Hu J, Liu Y, Lv Z, Li X, Qin X, Fan W (2011). Mortality and morbidity associated with simultaneous bilateral or staged bilateral total knee arthroplasty: a meta-analysis. Arch Orthop Trauma Surg.

[14] Husted H, Otte KS, Kristensen BB, Orsnes T, Wong C, Kehlet H (2010). Low risk of thromboembolic complications after fast-track hip and knee arthroplasty. Acta Orthop.

[15] Kahlenberg CA, Krell EC, Sculco TP, Katz JN, Nguyen JT, Figgie MP (2021). Differences in time to return to work among patients undergoing simultaneous versus staged bilateral total knee arthroplasty. Bone Joint J.

[16] Keenan AM, Tennant A, Fear J, Emery P, Conaghan PG (2006). Impact of multiple joint problems on daily living tasks in people in the community over age fifty-five. Arthritis Rheum.

[17] Lau RL, Perruccio AV, Gandhi R, Mahomed NN (2012). The role of surgeon volume on patient outcome in total knee arthroplasty: a systematic review of the literature. BMC Musculoskelet Disord.

[18] Liu L, Liu H, Zhang H, Song J, Zhang L (2019). Bilateral total knee arthroplasty: simultaneous or staged? A systematic review and meta-analysis. Medicine (Baltimore).

[19] Lorchaivej S, Suprasert P, Srisuwan T, Rujiwetpongstorn J (2022). Prevalence and risk factor of post-operative lower extremities deep vein thrombosis in patients undergoing gynecologic surgery: a single-institute cross-sectional study. Thromb J.

[20] Makaram NS, Roberts SB, Macpherson GJ (2021). Simultaneous bilateral total knee arthroplasty is associated with shorter length of stay but increased mortality compared with staged bilateral total knee arthroplasty: a systematic review and meta-analysis. J Arthroplasty.

[21] Metcalfe AJ, Stewart C, Postans N, Dodds AL, Holt CA, Roberts AP (2013). The effect of osteoarthritis of the knee on the biomechanics of other joints in the lower limbs. Bone Joint J.

[22] Parvizi J, Rasouli MR (2012). Simultaneous-bilateral TKA: double trouble—affirms. J Bone Joint Surg Br.

[23] Peat G, Thomas E, Wilkie R, Croft P (2006). Multiple joint pain and lower extremity disability in middle and old age. Disabil Rehabil.

[24] Rolfson O, Digas G, Herberts P, Kärrholm J, Brogström F, Garellick G (2014). One-stage bilateral total hip replacement is cost-saving. Orthop Musc Syst Curr Res.

[25] Schraknepper J, Dimitriou D, Helmy N, Hasler J, Radzanowski S, Flury A (2020). Influence of patient selection, component positioning and surgeon's caseload on the outcome of unicompartmental knee arthroplasty. Arch Orthop Trauma Surg.

[26] Seol JH, Seon JK, Song EK (2016). Comparison of postoperative complications and clinical outcomes between simultaneous and staged bilateral total knee arthroplasty. J Orthop Sci.

[27] Sheth DS, Cafri G, Paxton EW, Namba RS (2016). Bilateral simultaneous vs staged total knee arthroplasty: a comparison of complications and mortality. J Arthroplasty.

[28] Sobh AH, Siljander MP, Mells AJ, Koueiter DM, Moore DD, Karadsheh MS (2018). Cost analysis, complications, and discharge disposition associated with simultaneous vs staged bilateral total knee arthroplasty. J Arthroplasty.

[29] Tsay EL, Grace TR, Vail T, Ward D (2019). Bilateral simultaneous vs staged total knee arthroplasty: minimal difference in perioperative risks. J Arthroplasty.

[30] Tsiridis E, Pavlou G, Charity J, Tsiridis E, Gie G, West R (2008). The safety and efficacy of bilateral simultaneous total hip replacement: an analysis of 2063 cases. J Bone Joint Surg Br.

[31] Vanbiervliet J, Dobransky J, Poitras S, Beaule PE (2020). Safety of single-stage bilateral versus unilateral anterior total hip arthroplasty: a propensity-matched cohort study. J Bone Joint Surg Am.

[32] Yoon HS, Han CD, Yang IH (2010). Comparison of simultaneous bilateral and staged bilateral total knee arthroplasty in terms of perioperative complications. J Arthroplasty.

